# Application of Zero-Inflated Poisson Mixed Models in Prognostic Factors of Hepatitis C

**DOI:** 10.1155/2013/403151

**Published:** 2013-10-01

**Authors:** Alireza Akbarzadeh Baghban, Asma Pourhoseingholi, Farid Zayeri, Ali Akbar Jafari, Seyed Moayed Alavian

**Affiliations:** ^1^Department of Basic Sciences, School of Rehabilitation, Shahid Beheshti University of Medical Science, Tehran, Iran; ^2^Department of Biostatistics, School of Paramedical Science, Shahid Beheshti University of Medical Science, Tehran, Iran; ^3^Proteomics Research Center, School of Paramedical Science, Shahid Beheshti University of Medical Science, Tehran, Iran; ^4^Department of Statistics, Yazd University, Yazd, Iran; ^5^Baqiyatallah Research Center for Gastroenterology and Liver Diseases, Baqiyatallah University of Medical Sciences, Tehran, Iran

## Abstract

*Background and Objectives*. In recent years, hepatitis C virus (HCV) infection represents a major public health problem. Evaluation of risk factors is one of the solutions which help protect people from the infection. This study aims to employ zero-inflated Poisson mixed models to evaluate prognostic factors of hepatitis C. *Methods*. The data was collected from a longitudinal study during 2005–2010. First, mixed Poisson regression (PR) model was fitted to the data. Then, a mixed zero-inflated Poisson model was fitted with compound Poisson random effects. For evaluating the performance of the proposed mixed model, standard errors of estimators were compared. *Results*. The results obtained from mixed PR showed that genotype 3 and treatment protocol were statistically significant. Results of zero-inflated Poisson mixed model showed that age, sex, genotypes 2 and 3, the treatment protocol, and having risk factors had significant effects on viral load of HCV patients. Of these two models, the estimators of zero-inflated Poisson mixed model had the minimum standard errors. *Conclusions*. The results showed that a mixed zero-inflated Poisson model was the almost best fit. The proposed model can capture serial dependence, additional overdispersion, and excess zeros in the longitudinal count data.

## 1. Introduction

In recent years, hepatitis C virus (HCV) infection has been a major cause of liver diseases worldwide and represents a major public health problem [1–5]. Transfusion and contact with infected blood and its products, intravenous drug use, and contamination during medical procedures are among different risk factors of HCV [6–8]. An estimated 130–170 million people worldwide are infected with hepatitis C. The global prevalence of this infection is approximately 0.2%−40% [2, 9]. But there is a difference between developed and undeveloped countries in its prevalence. It is due to difference in health policies and medical care [10]. Apart from few studies that have been done on high-risk groups or in specific locations, no comprehensive and accurate estimate of HCV infection is available in Iran. According to two available studies which examined Iranian population, the prevalence of HCV infection in the general population is less than 1% [11, 12].

Hepatitis C is a common infection that causes chronic liver disease in the world [13]. The occurrence of end-stage liver disease caused by HCV is estimated to peak around 2020 [10, 14]. According to other studies, HCV infection is responsible for 20% of acute hepatitis cases, 70% of all chronic hepatitis cases, 40% of all cases of liver cirrhosis, 60% of hepatocellular carcinomas (HCC), and 30% of liver transplants [15]. 

In the coming decades, it is expected that the economic burden and mortality associated with hepatitis C rise [7, 16]. Unfortunately, the majority of infections do not respond to treatment and lead to chronic diseases. So it seems that controlling HCV infection is an important issue in public health [5, 17]. Risk factor evaluation in order to reduce the problem in the community is one solution to protect people from the infection.

In medical researches statistical modeling is a powerful approach in risk factor evaluation, but selection of good and appropriate model is important. When the response variable is count, there are some models that they use for analyzing such data. Sometimes count data have an overdispersion problem because of having large number of zeros. This phenomenon is called zero-inflation. Using usual count model in zero-inflated data causes misleading results.

Lambert [18] proposed the zero-inflated Poisson (ZIP) regression model for independent count data. For clustered count data, ZIP models have been developed, and different types of such models have been introduced and used in different studies [19, 20]. In this study, the relationship between 3 viral loads of each HCV patient and some risk and demographic factors was investigated using mixed ZIP regression. Details of mixed ZIP modeland its parameter estimation are described in [21, 22].

## 2. Methodology

### 2.1. Patient Selection

This is a longitudinal study and all data for this research were drawn from medical records of 186 patients with hepatitis C. All of these patients had been referred to Tehran hepatitis clinic, a clinic of Baqiyatallah Research Center for Gastroenterology and Liver Diseases, from 2005 to 2010. The Information concerning 186 patients includes viral load (HCV-RNA). The viral load had been recorded before the treatment, during the period of treatment, immediately after this period and 3 to 4 months after the end of the treatment. The viral load before treatment has been considered for baseline adjusting. The variables included in the study are as follows: demographic information including sex and age, genotypes including genotypes 1, 2 and 3, treatment protocol including combination therapy of standard Interferon (3 MU three times a week) plus Ribavirin (800–1200 mg per day) for 24 weeks or 48 weeks [23–25] as well as combination therapy of Peg-Interferon (Alfa 2a in a fixed dose of 180 micrograms per week) plus Ribavirin (800–1200 mg per day) for 24 weeks or 48 weeks [24, 26], history of blood transfusion, addiction (IV drug user), and contaminated needle stick. All of these factors were extracted from the patient's medical records. Therefore, five covariates including age, sex, genotype, protocol of treatment, and risk factor were entered in this study. Finally, 558 viral loads of HCV and their related information were extracted; it means that each patient was examined three times (the first time was baseline). On the other hand, negative HCV-RNA is considered as being below 100 and it is taken as zero in the analyses. Generally, HCV-RNA of 100 to 200,000 is considered as being very low; 200,000 to 1,000,000 as low; 1,000,000 to 5,000,000 as medium; 5,000,000 to 25,000,000 as high; and above 25,000,000 as very high.

### 2.2. Statistical Analysis

Descriptive statistics and frequency distribution such as mean, standard deviation, and percentage were calculated according to standard methods. The outcome variable of interest is the viral load of HCV patients. For calculating the viral load of HCV patients, where data are clustered on the subjects, a mixed ZIP model was employed. This model is a combination of zero-inflated and random effects models to control both zero-inflated and cluster structure of data [22]. On the other hand, the Poisson random effects model, without considering zero inflated structure of data, was carried out [27]. These two models were compared using standard error of their estimators. Significance was defined as *P* < 0.05. Stata 11 and R 2.13.1 program, were used for the analysis. 

## 3. Results

55 patients of the total 186 patients who were entered into this study were females. The mean and standard deviation of age were 42.88 and 11.17 years, respectively. Their age ranged between 19 and 76 years.  Table 1 shows the distribution of covariates in this study. Each patient had four viral loads for evaluating the treatment process.  Table 2 shows the distribution of six groups of viral load in 186 patients repeated four times for each. According to these results, 55.2% of patients had negative HCV-RNA, which means that zero inflated models is needed. At the first stage, PR regression with random effects (mixed PR) was fitted. The random effect was entered into this model for adjusting the clustered data structure. According to the results of this model, genotype 3 and treatment protocol were statistically significant.  Table 3 shows the results of this model. The significant Pearson Chi square goodness of fit (GOF) test (*P* < 0.001) along with other features of the model fit indicated that the mixed PR model produced a poor fit. On the other hand, a significant likelihood ratio test (*P* < 0.001) of dispersion statistic from zero showed that overdispersion has occurred in this data.

 In the next stage, ZIP model with random effects (mixed ZIP) was carried out to account for both clustering and excessive zeros. The covariates of age, sex, and genotype, protocol of treatment, and risk factors had significant effects on developing HCV-RNA at *α* = 0.05. The rate of virological response was higher in younger males. Subjects who had none of the risk factors, including the history of blood transfusion, addiction (IV drug user), and needle stick, were more likely to have virological response than others. Patients with genotype 3 and genotype 2 tended to have more virological response than those with genotype 1. The rate of virological response was also higher in subjects with combination therapy of Peg-Interferon plus Ribavirin. In addition to regression parameter in this model, two parameters of the random effects were estimated. The first estimate of random effect model (*ρ* = 0.3543) indicated the longitudinal correlation between the subjects. Also this random effect shows that the recurrence of the HCV-RNA every time partly depends on its value at the previous count. The second random effects (*τ* ^2^ = 1.61) indicated that the variation of data is much greater than the one shown by the first random effect. The results of this model are shown in Table 4. The comparison of these two models is presented in Table 5. The standard errors for covariate effects obtained from ZIP model were generally smaller than those obtained from PR regression with random effects.

## 4. Discussion

In this paper, a mixed ZIP model was used for clustered count data with excessive zero. Its results were compared with those of mixed PR model. 

In mixed ZIP model, all covariates had significant effects on the response variable. In this research, the rate of low viral load in men was more than that in women. The results of the studies done recently on patients with genotype 1 indicate that SVR in men is 2.5 times higher than that in women [28]. In the present study, this rate was 2.7 times as much. It seems that this difference is because of some physiological and psychological differences between men and women in the society. Also, patients with genotypes 3 and 2 had more virological response than patients with genotype 1. The fact that achieving SVR in genotype 1 is more difficult than in other genotypes has also been confirmed by the results of other studies [29]. On the other hand, risk factors decrease the rate of virological response. It seems that such results have been obtained due to the relationship that exists between the risk factors and the genotype. For example, there is a direct relationship between genotype 1 and injecting drug users, blood transfusion, and contact with infected blood as well as its products [30]. Two main protocols of treatment were used in this study based on the genotype of patients. According to the results, combination therapy of Peg-Interferon plus Ribavirin had better results than combination therapy of standard interferon plus Ribavirin. The large number of studies which have been conducted so far showed that Peg-Interferon plus Ribavirin had been most responsive to treatment [31–34]. So it seems that this protocol has been the best choice [35, 36]. Unfortunately in Iran, due to high cost of the drug, it is not the first choice for doctors. Usually when patients did not respond to the treatment, doctors decided to prescribe Peg-Interferon plus Ribavirin [36].

Although clustered count data with extra zeros often occur, few methods have been developed for correlated data with extra zeros [37]. There are some studies done on the extension of zero inflated models in order to accommodate random effects [20, 38]. In all of these models, there were two separate random effects in the models; therefore, the interpretation of the results was more difficult and sometimes confusing. A mixed ZIP model that was used in this paper has been introduced by Ma et al. in 2009. This proposed model had a compound Poisson random effect structure. This distribution was very useful for characterizing both the excessive zeros and clustering structure of the data. Another advantage of this model was its computational efficiency which was highly useful for analyzing massive data sets [21]. In this data set, the programs run after two minutes and thirty seconds. A comparison was also made between the results gained by this model and those gained by one of the standard methods for analysis of longitudinal count data (mixed PR model). The standard errors for covariate effects obtained from the mixed ZIP model were generally smaller than those obtained from mixed PR model. Results were compared by standard error, because there is not any goodness of fit criterion for mixed ZIP model yet. Standard errors get larger unless the extra zeros are accounted for. If the excessive number of zeros is not adjusted, the standard deviation gets larger. Therefore, the standard deviation of estimators in ZIP model is smaller than that of the other model. Since the zero inflated structure has not been taken into account in the other model, the standard deviation of estimators gets larger compared to ZIP model. 

In conclusion, the mixed zero inflated Poisson models were seen as almost being the best fit. As with this research, clustered zero inflated count data is quite frequent in medical researchers. Since a wrong model would yield unreliable results, therefore, choosing the best and correct model for analyzing the data is highly important. 

## Figures and Tables

**Table 1:** The distribution of covariates in this study.

Variable	Category	*n*	%
Sex	Female	55	29.6
	Male	131	70.4
Risk factor	Yes	104	55.9
	No	82	44.1
Genotype	1	142	76.3
	2	4	2.2
	3	40	21.5
Treatment	Interferon plus Ribavirin	100	53.8
	Peg-Interferon plus Ribavirin	86	46.2

**Table 2:** The distribution of six groups of viral load in 186 patients.

Viral load	Male		Female		Total	
	*n*	%	*n*	%	*n*	%
Zero	289	55.2	116	52.7	405	54.5
200 to 200,000	78	14.9	36	16.4	114	15.3
200,000 to 1,000,000	110	21	45	20.5	155	20.8
1,000,000 to 5,000,000	40	7.6	21	9.5	61	8.2
5,000,000 to 25,000,000	7	1.3	1	0.5	8	1.1
Above 25,000,000	0	0	1	0.5	1	0.1

Total	524	100	220	100	744	100

**Table 3:** The results of mixed PR model.

Parameter	Category	Estimated	Standard error	*P*-value
Intercept		0.7320	0.188	<0.001
Age		0.0049	0.0036	0.195
Sex	Female*			
	Male	−0.0487	0.0864	0.580
Riskfactor	No*			
	Yes	0.154	0.0803	0.053
Genotype	1*			
	2	−0.1760	0.2785	0.539
	3	−0.2357	0.0984	0.013
Treatment	Interferon plus Ribavirin*			
	Peg-Interferon plus Ribavirin	−0.2584	0.0728	0.001
Variance component		0.2469		<0.001

*Reference group.

**Table 4:** The results of mixed ZIP model.

Parameter	Category	Estimated	Standard error	*P*-value
Intercept		3.6517	0.1659	<0.001
Age		0.0046	0.0023	0.037
Sex	Female*			
	Male	−0.978	0.0833	<0.001
Risk factor	No			
	Yes	0.4626	0.074	<0.001
Genotype	1*			
	2	−0.8006	0.2381	<0.001
	3	−1.148	0.0932	<0.001
Treatment	Interferon plus Ribavirin*			
	Peg-Interferon plus Ribavirin	−0.6628	0.0737	<0.001
*λ*		0.6891		
*τ* ^2^		1.6153		
*ρ*		0.3634		

*Reference group.

**Table 5:** The comparison of mixed ZIP and mixed PR.

Parameter	Category	Mixed ZIP model		Mixed PR model	
		Estimated	Standard error	Estimated	Standard error
Intercept		3.6517	0.1659	0.7320	0.188
Age		0.0046	0.0023	0.0049	0.0036
Sex	Female*				
	Male	−0.978	0.0833	−0.0487	0.0864
Risk factor	No				
	Yes	0.4626	0.074	0.154	0.0803
Genotype	1*				
	2	−0.8006	0.2381	−0.1760	0.2785
	3	−1.148	0.0932	−0.2357	0.0984
Treatment	Interferon plus Ribavirin*				
	Peg-Interferon plus Ribavirin	−0.6628	0.0737	−0.2584	0.0728

*Reference group.

## References

[1] Alavian SM (2005). Are the real HCV infection features in Iranian patients the same as what is expected?. *Hepatitis Monthly*.

[2] Alter MJ (2007). Epidemiology of hepatitis C virus infection. *World Journal of Gastroenterology*.

[3] Alavian SM (2010). Hepatitis C virus infection: epidemiology, risk factors and prevention strategies in public health in I.R.IRAN. *Gastroenterology and Hepatology from Bed to Bench*.

[4] Alavian S-M (2011). New globally faces of hepatitis B and C in the world. *Gastroenterology and Hepatology from Bed to Bench*.

[5] Alavian S-M, Adibi P, Zali M-R (2005). Hepatitis C virus in Iran: epidemiology of an emerging infection. *Archives of Iranian Medicine*.

[6] Alavian SM, Bagheri-Lankarani K, Mahdavi-Mazdeh M, Nourozi S (2008). Hepatitis B and C in dialysis units in Iran: changing the epidemiology. *Hemodialysis International*.

[7] Alavian S-M (2008). We need a new national approach to control hepatitis C: it is becoming too late. *Hepatitis Monthly*.

[8] Alavian SM (2004). Optimal therapy for hepatitis C. *Hepatitis Monthly*.

[9] Alter MJ (1997). Epidemiology of hepatitis C. *Hepatology*.

[10] Alavian SM, Lankarani KB, Aalaei-Andabili SH (2011). Treatment of chronic hepatitis C infection: update of the recommendations from scientific leader’s meeting-28th july 2011-Tehran, IR Iran. *Hepatitis Monthly*.

[11] Alavian SM, Ahmadzad-Asl M, Lankarani KB, Shahbabaie MA, Ahmadi AB, Kabir A (2009). Hepatitis C infection in the general population of Iran: a systematic review. *Hepatitis Monthly*.

[12] Merat S, Rezvan H, Nouraie M (2010). Seroprevalence of hepatitis C virus: the first population-based study from Iran. *International Journal of Infectious Diseases*.

[13] Touzet S, Kraemer L, Colin C, Pradat P, Lainor D, Baily F (2000). Epidemiology of hepatitis C virus infection in seven European Union countries: a critical analysis of the litreature. HENCORE Group. Hepatitis C European Network for Co-operative Resarch. *European Journal of Gastroenterology & Hepatology*.

[14] Wyles DL (2010). Moving beyond interferon alfa: investigational drugs for hepatitis C virus infection. *Topics in HIV Medicine*.

[15] Ahmadipour MH, Alavian SM, Amini S, Azadmanesh K (2003). Hepatitis C virus genotypes. *Hepatitis Monthly*.

[16] Brown RS, Caglio PJ (2003). Scope of worldwide hepatitis C problem. *Liver Transplantation*.

[17] Hwang S-J, Lee S-D, Lu R-H (2001). Hepatitis C viral genotype influences the clinical outcome of patients with acute posttransfusion hepatitis C. *Journal of Medical Virology*.

[18] Lambert D (1992). Zero-inflated poisson regression, with an application to defects in manufacturing. *Technometrics*.

[19] Böhning D, Dietz E, Schlattmann P, Mendonça L, Kirchner U (1999). The zero-inflated Poisson model and the decayed, missing and filled teeth index in dental epidemiology. *Journal of the Royal Statistical Society A*.

[20] Hall DB (2000). Zero-inflated poisson and binomial regression with random effects: a case study. *Biometrics*.

[21] Ma R, Hasan MT, Sneddon G (2009). Modelling heterogeneity in clustered count data with extra zeros using compound Poisson random effect. *Statistics in Medicine*.

[22] Hasan MT, Sneddon G, Ma R (2009). Pattern-mixture zero-inflated mixed models for longitudinal unbalanced count data with excessive zeros. *Biometrical Journal*.

[23] Myers RP, Regimbeau C, Thevenot T (2002). Interferon for interferon naive patients with chronic hepatitis C. *Cochrane Database of Systematic Reviews*.

[24] Poynard T, Yuen M-F, Ratziu V, Lung Lai C (2003). Viral hepatitis C. *The Lancet*.

[25] Poynard T, Mchutchison J, Goodman Z, Ling M-H, Albrecht J (2000). Is an “a la carte” combination interferon alfa-2b plus ribavirin regimen possible for the first line treatment in patients with chronic hepatitis C?. *Hepatology*.

[26] Fried MW, Shiffman ML, Rajender Reddy K (2002). Peginterferon alfa-2a plus ribavirin for chronic hepatitis C virus infection. *The New England Journal of Medicine*.

[27] Agresti A (2007). *An Introduction to Categorical Data Analysis*.

[28] Tsubota A, Shimada N, Yoshizawa K (2012). Contribution of ribavirin transporter gene polymorphism to treatment response in peginterferon plus ribavirin therapy for HCV genotype 1b patients. *Liver International*.

[29] Ionita-Radu F, Rascanu A, Cheiab B (2011). IL28B polymorphism—predictive factor of HCV infected genotype 1 individuals to treatment response and management of therapy. *Romanian Journal of Internal Medicine*.

[30] Samimi-Rad K, Nategh R, Malekzadeh R, Norder H, Magnius L (2004). Molecular epidemiology of hepatitis C virus in Iran as reflected by phylogenetic analysis of the NS5B region. *Journal of Medical Virology*.

[31] Ferenci P, Fried MW, Shiffman ML (2005). Predicting sustained virological responses in chronic hepatitis C patients treated with peginterferon alfa-2a (40 KD)/ribavirin. *Journal of Hepatology*.

[32] Manns MP, McHutchison JG, Gordon SC (2001). Peginterferon alfa-2b plus ribavirin compared with interferonalfa-2b plus ribavirin for initial treatment of chronic hepatitis C: a randomised trial. *The Lancet*.

[33] Hadziyannis SJ, Sette H, Morgan TR (2004). Peginterferon-*α*2a and ribavirin combination therapy in chronic hepatitis C: a randomized study of treatment duration and ribavirin dose. *Annals of Internal Medicine*.

[34] Strader DB, Wright T, Thomas DL, Seeff LB (2004). Diagnosis, Management, and Treatment of Hepatitis C. *Hepatology*.

[35] Farrell GC (2007). New hepatitis C guidelines for the Asia-Pacific region: APASL consensus statements on the diagnosis, management and treatment of hepatitis C virus infection. *Journal of Gastroenterology and Hepatology*.

[36] Shiffman ML, Suter F, Bacon BR (2007). Peginterferon alfa-2a and ribavirin for 16 or 24 weeks in HCV genotype 2 or 3. *The New England Journal of Medicine*.

[37] Lam KF, Xue H, Bun Cheung Y (2006). Semiparametric analysis of zero-inflated count data. *Biometrics*.

[38] Min Y, Agresti A (2005). Random effect models for repeated measures of zero-inflated count data. *Statistical Modelling*.

